# Discovering Karima (Euphorbiaceae), a New Crotonoid Genus from West Tropical Africa Long Hidden within Croton

**DOI:** 10.1371/journal.pone.0152110

**Published:** 2016-04-06

**Authors:** Martin Cheek, Gill Challen, Aiah Lebbie, Hannah Banks, Patricia Barberá, Ricarda Riina

**Affiliations:** 1 Science Department, Royal Botanic Gardens, Kew, Surrey, United Kingdom; 2 National Herbarium of Sierra Leone, Dept. of Biological Sciences, Njala University, PMB, Freetown, Sierra Leone; 3 Department of Biodiversity and Conservation, Real Jardín Botánico, RJB-CSIC, Plaza de Murillo, Madrid, Spain; University of Florida, UNITED STATES

## Abstract

*Croton scarciesii* (Euphorbiaceae-Crotonoideae), a rheophytic shrub from West Africa, is shown to have been misplaced in *Croton* for 120 years, having none of the diagnostic characters of that genus, but rather a set of characters present in no known genus of the family. Pollen analysis shows that the new genus *Karima* belongs to the inaperturate crotonoid group. Analysis of a concatenated molecular dataset combining *trnL-F* and *rbcL* sequences positioned *Karima* as sister to *Neoholstia* from south eastern tropical Africa in a well-supported clade comprised of genera of subtribes Grosserineae and Neoboutonieae of the inaperturate crotonoid genera. Several morphological characters support the relationship of *Karima* with *Neoholstia*, yet separation is merited by numerous characters usually associated with generic rank in Euphorbiaceae. Quantitative ecological data and a conservation assessment supplement illustrations and descriptions of the taxon.

## Introduction

The environmental impact assessment of the Bumbuna-Yiben Hydroelectric Dam project in Sierra Leone which was led by Xander van der Burgt of the Royal Botanic Gardens, Kew, with colleagues from the National Herbarium of Sierra Leone provided numerous plant collections [1]. Among the resultant herbarium specimens, one (*Momoh* 94) proved of great interest. *Momoh* 94 clearly represents a member of the Euphorbiaceae since its fruits bear three, bifurcate, persistent styles and are tricoccal, dehiscing septicidally, leaving a central vascular column. The presence of a single ovule in each carpel excludes the material from Phyllanthaceae [2, 3], as the presence of petals and absence of white exudate excludes the material from Euphorbiaceae–Euphorbioideae [2, 3]. However, although it was eventually matched at K with material named as *Croton scarciesii* Scott-Elliot, including both syntypes, these authors noted that the main morphological characters of *Croton scarciesii* disagree with most typical *Croton* L. features and matched no known genus. Independently at MA, in the context of a taxonomic revision of African *Croton*, two of the coauthors arrived at the same conclusion. Using evidence from morphology, pollen, molecular phylogenetics and geography, we describe and illustrate a new genus, erecting the name *Karima* and place it within Euphorbiaceae-Crotonoideae. We compare the new monotypic genus *Karima* to *Croton* and to its closest relative in a molecular phylogeny including a subset of other inaperturate crotonoid taxa. A complementary field study of the ecology of *Karima* was also conducted at the Taia River in Sierra Leone. Despite recent phylogenetic and taxonomic studies, our finding highlights the still problematic classification of Euphorbiaceae, with many poorly known species, issues with generic delimitation, and new genera being described.

## Materials and Methods

### Ethics statement

*Momoh* 94, which triggered this paper, was collected during field studies for an Environmental Impact assessment of the Bumbuna hydroelectric dam, as part of a survey co-ordinated and managed by the international environmental consultancy company ERM for the engineering company Joule Africa. The National Herbarium of Sierra Leone, Njala University, issued a permit to collect herbarium specimens in this context on 9^th^ May 2014 to the leader of the botanical studies, van der Burgt. A further permit to export herbarium specimens was issued on 3^rd^ June 2014.

Field studies of *Karima* at the Taia River reported in S2 Appendix were conducted by the National Herbarium of Sierra Leone, which has the national statutory responsibility for study of the vegetation and plant species of Sierra Leone and requires no individual permits in order to conduct field surveys.

The area of the Bumbuna hydroelectric project is controlled by the state of Sierra Leone. The study area of the Taia river at Njala University is not privately owned, nor protected. *Karima scarciesii* (Synonym *Croton scarciesii*) is not a protected species.

### Pollen study

Pollen morphology has been traditionally used in Euphorbiaceae taxonomy for delimitation of infrafamilial taxa [3]. Pollen samples were collected from *Momoh* 94 (FBC, K). Whole, unacetolysed, dehisced anthers from the herbarium specimen were placed on a stub and sputter-coated with platinum in a Quorom Q150T coater and examined in a Hitatchi 54700 scanning electron microscope.

### Taxon sampling and molecular data

Guided by the results from pollen morphology, we limited our taxon sampling for phylogenetic analyses to the two clades of inaperturate crotonoids (“C1” and “C2” sensu Wurdack et al. ([2]: figure 4). Based on the results of a preliminary Maximum Parsimony phylogenetic analysis (data not shown) of all sequences of inaperturate crotonoids included in [2] using PAUP* v.4.0b.10 [4], our final molecular data matrix consisted of all (26) species of clade C2 of the combined analysis of Wurdack et al (2005), three representatives from clade C1, and newly generated sequences of *Neoholstia tenuifolia* and two accessions of *Karima scarciesii* (*Momoh* 94, Sierra Leone and *Jongkind* 4208, Cote D’Ivoire). A non-crotonoid species, *Nealchornea yapurensis* Huber (Euphorbioideae), was also included as the most distant outgroup. A complete list of accessions, including newly generated sequences and their respective Genbank numbers, are listed in S1 Appendix.

For *Karima scarciesii* and *Neoholstia tenuifolia* samples, DNA extraction, polymerase chain reaction (PCR) amplification, and sequencing followed the laboratory procedures described in [5]. For the region comprising the *trnL* intron and *trnL-F* intergenic spacer (hereafter referred to as “*trnL-F”*) we used the primers indicated in [2]. The *rbcL* region was partially sequenced (first half) using primers rbcL1F (ATG-TCA-CCA-CAA-ACA-GAR-AC) and rbcL724R (TCG-CAT-GTA-CCC-TGC-AGT-TGC). The new sequences were assembled and edited using Staden Package v.2003.0b1 [6] and were deposited in GenBank (S1 Appendix). Previously published sequences were obtained from NCBI GenBank (www.ncbi.nlm.nih.gov). Sequences were aligned manually with MacClade v.4.08a [7], following the similarity criterion as suggested by [8]. Alignment of the *rbcL* data was straightforward and gap-free, whereas the *trnL-F* matrix required the inclusion of several gaps. The final alignment of each matrix (*rbcL*, *trnL-F*) was end-trimmed to remove most of the characters with missing data. The two matrices were combined into a single data set with two partitions corresponding to each molecular marker (*rbcL*, *trnL-F*). The combined matrix is available at TreeBase (http://purl.org/phylo/treebase/phylows/study/TB2:S18891).

### Phylogenetic analyses

The concatenated matrix with two data partitions was analyzed using a Bayesian approach. MrModeltest v.3.7 [9] was used to estimate the most appropriate model of sequence evolution for each data partition under the Akaike Information Criterion (AIC) [10]. The HKY+G and GTR+I+G models were selected for the *trnL-F* and *rbcL* datasets, respectively.

Bayesian inference, based on a Markov Chain Monte Carlo (MCMC) approach [11], was conducted in MrBayes v.3.2.1 [12]. Base equilibrium frequencies, instantaneous substitution rates, and among-site rate variation values were estimated independently for each partition on shared topologies. Two runs of 10 million generations were conducted, and trees were sampled every 1,000 generations. Each run consisted of four independent Markov chains initiated from a random starting tree and using the default temperature (0.2). The resultant Ln likelihood and model parameters from the MCMC runs were inspected using Tracer v.1.5 [13] to determine run convergence and stationarity as indicated by the effective sample size (ESS) values, which should be higher than 100. One-fourth of the MCMC samples from each run was discarded as “burn-in.” The remaining trees were pooled into a 50% majority rule consensus tree with clade credibility values. The consensus tree was visualized and edited in FigTree v.1.3.1. Five hundred maximum likelihood (ML) [14] bootstrap replicates, using the model GTR + G and the same data partitions as in the Bayesian analysis above, were implemented in RAxML v.7.0.3 [15] to generate an additional measure of clade support.

### Taxonomic treatment

A comparative morphological study of the new taxon was conducted using collections from the following herbaria: B, BM, BR, E, FBC, FHO, GC, IFAN, K, LISC, MO, P, SL, UCJ, US, WAG, Z. Codes for cited herbaria follow Index Herbariorum [16]. Cited specimens which have been seen by one or more author are annotated “!”, prefixed by a seven digit number for those with barcodes. The overall morphology was documented, described and illustrated following botanical standard procedures as documented in [17]. Information about habit, habitat, and distribution was taken from specimen labels and field observations. Specimens and protologues of all inaperturate Euphorbiaceae genera, and especially genera indicated as being closely related to *Karima* according to the phylogenetic results, were studied and compared with the new taxon. The conservation status was evaluated using IUCN criteria [18].

### Ecology

Field studies were conducted in the Taia river of Sierra Leone in May 2015, before the main wet season so that plants were not totally submerged and so that there would be no risk of the fieldworkers being swept away by the currents. The plots were placed around patches of the study species to obtain quantitative data on plant densities, spacings and heights, which were measured by metre rules and tape measures. Identifications of *Karima* were confirmed at the National Herbarium of Sierra Leone at the University of Njala, and further confirmed at K using photographs of the study subjects. All photographs associated with this paper were taken during this study.

### Nomenclature

The electronic version of this article in Portable Document Format (PDF) in a work with an ISSN or ISBN will represent a published work according to the International Code of Nomenclature for algae, fungi, and plants [19], and hence the new names contained in the electronic publication of a PLOS article are effectively published under that Code from the electronic edition alone, so there is no need to provide printed copies.

In addition, the new names contained in this work have been submitted to IPNI, from where they will be made available to the Global Names Index. The IPNI LSIDs can be resolved and the associated information viewed through any standard web browser by appending the LSID contained in this publication to the prefix http://ipni.org/. The online version of this work is archived and available from the following digital repositories: PubMed Central, LOCKSS.

## Results

### Pollen description

**Fig 1:**
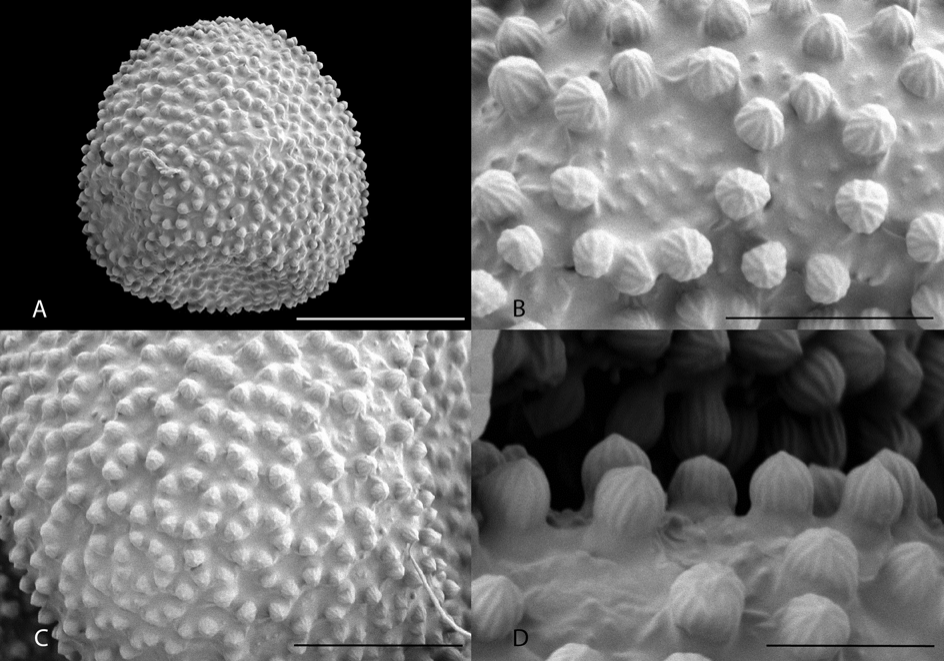
SEM micrographs of pollen of *Karima scarciesii*. A. whole grain, B. close-up of rings of subunits, C. surface of pollen grain, D. close-up of supratectal subunits, showing longitudinal ridges, and granulate lacunae base. All from *Momoh* 94. Scale bars, 20 μm (A), 5 μm (B), 10 μm (C), 3 μm (D).

The pollen grains are spheroidal, inaperturate and have a crotonoid exine sculpture pattern with supratectal subunits attached to the upper edge of low, circular muri each enclosing a central lacuna. The diameter of the grains is (35–)38–40 μm as measured by SEM from a sample of 20 pollen grains. The subunit rings are (2.8–)3.5–4(–5.2) μm diam., with (5–)7(–9) subunits per ring. Subunits are ovoid-spheroidal, 1–1.3 x 1–1.5 μm, strongly longitudinally ridged, with 12–14 ridges arising from the base, several of which unite with each other before converging at the obtuse apex. The floors of the central lacunae each have 7–20 scattered granules, each c. 0.1 μm diam. (Fig 1).

### Molecular phylogeny

**Fig 2:**
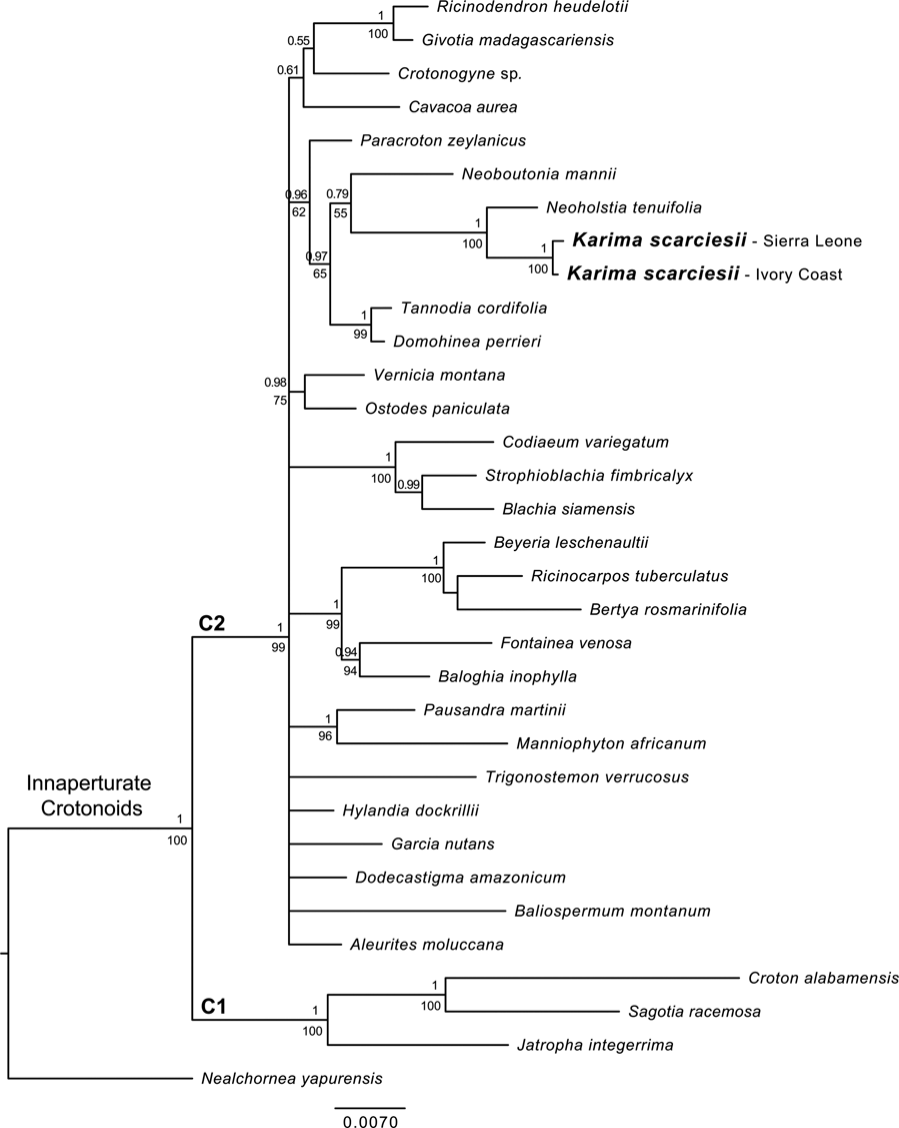
Bayesian consensus tree from analysis of concatenated dataset combining *trnL-F* and *rbcL* data showing the position of *Karima scarciesii* in the inaperturate crotonoids. Bayesian posterior probabilities and Maximum likelihood bootstrap values (> 0.5 or 50%) are shown above and below branches, respectively. Clades C1, C2 and inaperturate crotonoids according to Wurdack et al. [2].

The concatenated dataset combining *trnL-F* and part of *rbcL* contained 33 accessions and 2728 aligned positions, of which 202 were parsimony-informative. In the case of *rbcL* we were only able to amplify part of this region for the newly sampled taxa, therefore we only used the corresponding fragment of the published sequences downloaded from Genbank. The *trnL-F* dataset with 1365 aligned positions has more parsimony-informative characters (118) than the *rbcL* data set with 666 aligned positions and 84 parsimony-informative characters. The *rbcL* fragments for the two accessions of *Karima scarciesii* are identical. The two *trnL-F* sequences are nearly identical and differ only in one position, however, one of the accessions (*Jongkind* 4208) has 55 characters missing at the beginning of the sequence. Preliminary Bayesian analyses of the individual datasets did not show evidence of topological incongruence between the two genetic markers (results not shown). Given the lack of obvious conflict between the topologies resulting from individual matrices, and following the same strategy as [2], we analyzed the combined dataset (*rbcL* + *trnL-F*). The Bayesian consensus tree (Fig 2) resulting from the analysis of the concatenated matrix shows a phylogenetic structure congruent with the phylogenetic analyses of [2] (their figures 1, 2, 3). Representatives of clades C1 and C2 are sister groups with 100% Bayesian posterior probability (Fig 2). As in [2], clade C2 is highly supported but resolution at the backbone is quite low.

The two accessions of *Karima scarciesii* are recovered as an exclusive lineage (100% Bayesian posterior probability, BPP). The genus *Karima* is part of a highly supported clade (96% BPP), which also includes representatives of genera *Domohinea* Leandri, *Neoboutonia* Müll.Arg., *Neoholstia* Rauschert, *Paracroton* Miq., and *Tannodia* Baill. The new species is most closely related to *Neoholstia tenuifolia* (Pax) Rauschert (100% BPP), and sister to them but with low to moderate clade support is *Neoboutonia mannii* (79% BPP). The clade formed by *Domohinea perrieri* Leandri and *Tannodia cordifolia* Baill. is sister to the *Neoboutonia-Neoholstia-Karima* clade, and *Paracroton zeylanicus* (Müll.Arg.) N.P.Balakr. & Chakrab. is in a solitary branch sister to the two clades above (96% BPP) (Fig 2).

### Morphological differences

The morphological differences of *Karima* from *Croton sensu stricto* are extensive (Table 1). It is surprising that no other researchers have remarked upon the absence in *Croton scarciesii* (that is, the new genus, *Karima*) of the key diagnostic characters of *Croton*, such as lepidote scales or stellate hairs, the presence of nectary glands at the junction of the petiole and leaf blade, a crotonoid inflorescence (thyrsoid inflorescence with female and male flowers at base, male towards apex), and inflexed staminal filaments in flower buds.

**Table 1:** The characters separating *Croton* from *Karima* (*Croton* characters taken from [20, 21, 22].

	*Croton*	*Karima*
Indumentum	Stellate &/or lepidote	Simple
Petiolar or basilaminar glands	Two or more glands usually conspicuous	Glandular tissue entirely absent from leaves.
Inflorescence structure	Inflorescences terminal on principal axes, bisexual, with 1-few female flowers at base of otherwise male inflorescence	Inflorescences never present on principal axes, terminal only on spur (lateral) shoots. Male inflorescences (multiple flowers) usually separate from female inflorescences (1-flowered)
Flower presentation	Flowers never concealed within bracts	Flowers concealed within concave bracts until immediately preceding anthesis
Staminal filaments position within bud	Inflexed	Erect
Receptacle	Stamens usually inserted on a raised, hairy receptacle	Receptacle neither raised nor hairy

The placement of *Karima* near *Neoholstia* was reached by molecular analysis but is strongly supported by morphology (Table 2). The two genera share stalked, glandular, red-apexed glands on the bracts (unusual in Crotonoideae), and leaf texture and venation are also similar, as is the general indumentum type (simple, erect, robust, translucent hairs). The tuberculate fruits of both genera are also similar, apart from differences in the styles. However there are major qualitative characters that divide the two taxa. These characters are usual in serving to distinguish genera, rather than species, in Euphorbiaceae as can be seen in [21]. These major qualitative characters concern differences in gross architecture, in sexuality, in inflorescence structure and placement, in presence/absence of both bud-scales and of leaf heteromorphy (Table 2).

**Table 2:** The characters separating *Karima* from *Neoholstia*. Data for *Neoholstia* taken from [23] and from observations on material at K.

	*Karima*	*Neoholstia*
Architecture	Distinct principal vertical (orthotropic) axis and short axillary horizontal (spur or plagiotropic) shoots	Stems not differentiated into orthotropic and plagiotropic shoots
Bud-scales	Bud-scales not present, buds (rarely seen) clad with dormant leaves	Abundant, large specialised bud-scales with rounded apices, persistent throughout the year
Stipules	Persistent throughout the year, conspicuous, glandular hairy, becoming indurated	“Minute, soon deciduous” [21]. Very inconspicuous and often not detectable
Leaf-blade	Monomorphic on each plant, rhombic, apex rounded	Usually polymorphic on one stem, larger leaves ovate, often 2–3 lobed, apex acuminate, base cordate, smaller leaves entire, base truncate
Sexuality	Monoecious	Dioecious
Inflorescence placement	Terminal on axillary shoots, absent from main axis	Terminal on all shoots
Female inflorescence	Inconspicuous, 1-flowered, concealed in spur leaves	Conspicuous raceme with numerous flowers
Male inflorescence	Flowers usually glomerulate, when in raceme, flowers numerous (>10) at each node, concealed within a naviculate bract until anthesis	Flowers always in long (13–17(–25) cm long) raceme, flowers 1(–2) per node, bract not concealing the developing buds at any stage
Staminal filaments	Free to base	Connate at base
Geography	West Africa	SE Africa

### Ecology

The results of the field studies are in S2 Appendix.

## Discussion

### Former placement in *Croton*

*Karima* was erroneously misplaced in *Croton* as *Croton scarciesii* by Scott-Elliot [24]. The protologue is part of a paper [24] describing botanical novelties resulting from his participation in the survey of the boundary between Sierra Leone and the then French Guinea (now Republique de Guinée), also known as Guinea-Conakry. In that paper most of the species are described from his specimens by the corresponding family specialists at Kew. Where no family specialist was available, Scott-Elliot himself took on responsibility for placing and describing the taxa that he considered to be new to science. Evidently, at that time, Kew had no Euphorbiaceae specialist. The placement of the taxon in *Croton* appears never to have been challenged. Opportunities to detect the error were missed when the *Croton* accounts for the Flora of West Tropical Africa were written by the respective editors Hutchinson [25] and, in the revised edition, by Keay [26]. It is possible that they were under pressure of time to complete these accounts and so could not critically review Scott-Elliot's generic placement of this species. Perhaps they attributed the obviously anomalous morphology within *Croton* of the species to its rheophytic ecology. It is also possible that they did not dissect the flowers, which would have revealed additional characters that militated against placement in *Croton* (Table 1).

### Placement and establishment of *Karima* based on multiple evidence

Despite the strong morphological separation of *Karima* from *Croton* (Table 1), examination of the pollen indicates that grains are spheroidal, inaperturate and with crotonoid exine sculpturing. This characterises subfamily Crotonoideae, and together with the gross morphological characters of the indumentum, leaf and flower and utilisation of the keys in Radcliffe-Smith [21] and Webster [27], suggests that *Karima* is best placed in Crotonoideae in the region of Codiaeae and Aleuritidieae. The molecular phylogeny also confirms that *Karima* is part of Crotonoideae, and that it belongs to the inaperturate crotonoid clade C2 of Wurdack et al. [2]. This clade, emended in this paper (Fig 2), is comprised of all sampled members (27 genera) of the following tribes as delimited by Radcliffe-Smith [21]: Codiaeae (Pax) Hutch. (excepting *Ophellantha* Standl., placed in C1); Trigonostemeae G.L. Webster; Ricinocarpeae Muell. Arg.; Ricinodendreae (Pax) Hutch.; Aleuritideae Hurus (excepting *Sandwithia* Lanj., placed in C1), with the addition of *Paracroton* Miq., sister to the rest of the *Karima* clade and formerly placed doubtfully in Crotoneae by Radcliffe-Smith [21]. However 15 genera (c. one third) of those placed in these tribes by Radcliffe-Smith [21] did not appear in the combined data-set of Wurdack et al., although some of these have placements in the individual locus trees [2]. The topology of clade C2 may change if any of these genera are included in a future analysis.

Key to the putative genera of the *Karima* clade of C2 inaperturate Crotonoids (sensu Wurdack et al. [2]), modified from key to the genera of Grossinerinae G.L Webster (Radcliffe-Smith [21]: 334–335). Genera in **bold** did not appear in the phylogenetic analysis of the combined dataset in Wurdack et al. [2]

1 Indumentum stellate or lepidote, at least in part.......................................................................................2
1 Indumentum simple, or absent.....................................................................................3
2 Sap red, leaf-blades penninerved, petals present......................................................................*Paracroton*
2 Sap clear, leaf-blades palminerved, petals absent......................................................................*Neoboutonia*
3 Leaves mostly 3-plinerved; pollen echinate..........................................4
3 Leaves penninerved; pollen processes rounded to obtuse..........................6
4 Female sepals 2–3; male receptacle pilose..............................................................................*Domohinea*
4 Female sepals 4–5; male receptacle glabrous.....................................................................................5
5 Female petals exceeding the calyx...........................................*Tannodia*
5 Female petals exceeded by the calyx.......................................*Neoholstia*
6 Male petals glabrous; leaves pellucid-punctate...........................***Grossera***
6 Male petals pubescent; leaves not, or only sparingly pellucid-punctate.........7
7 Inflorescences terminal or subterminal; male disc dissected, seeds carunculate.................................................................................. *Karima*
7 Inflorescences axillary; male disc annular; seeds ecarunculate.......................................................................................8
8 Stamens 6–8; fruit tomentose..............................................***Tapoides***
8 Stamens >20; fruit glabrous..........................................***Anomalocalyx***

The DNA sequence data indicate that *Karima scarciesii* is most closely related to the southeastern tropical African *Neoholstia tenuifolia* (Fig 2). We did not include the new taxon within the monotypic genus *Neoholstia* [21] because morphological differences between the two taxa are considerable (see above and Table 2). Furthermore, the current phylogeny of the C2 clade of Wurdack et al. [2] is only sampled at the level of a single species per genus and so provides little resolution to assess generic delimitation with confidence. Phylogenetic analyses including additional species from each genus and using more informative molecular markers are still needed to better address taxonomic delimitation within clade C2 of the inaperturate crotonoids.

### Taxonomic treatment

*Karima* Cheek & Riina gen. nov. [urn:lsid:ipni.org: names:77153667–1]

Type: Karima scarciesii (Scott-Elliot) Cheek

**Fig 3:**
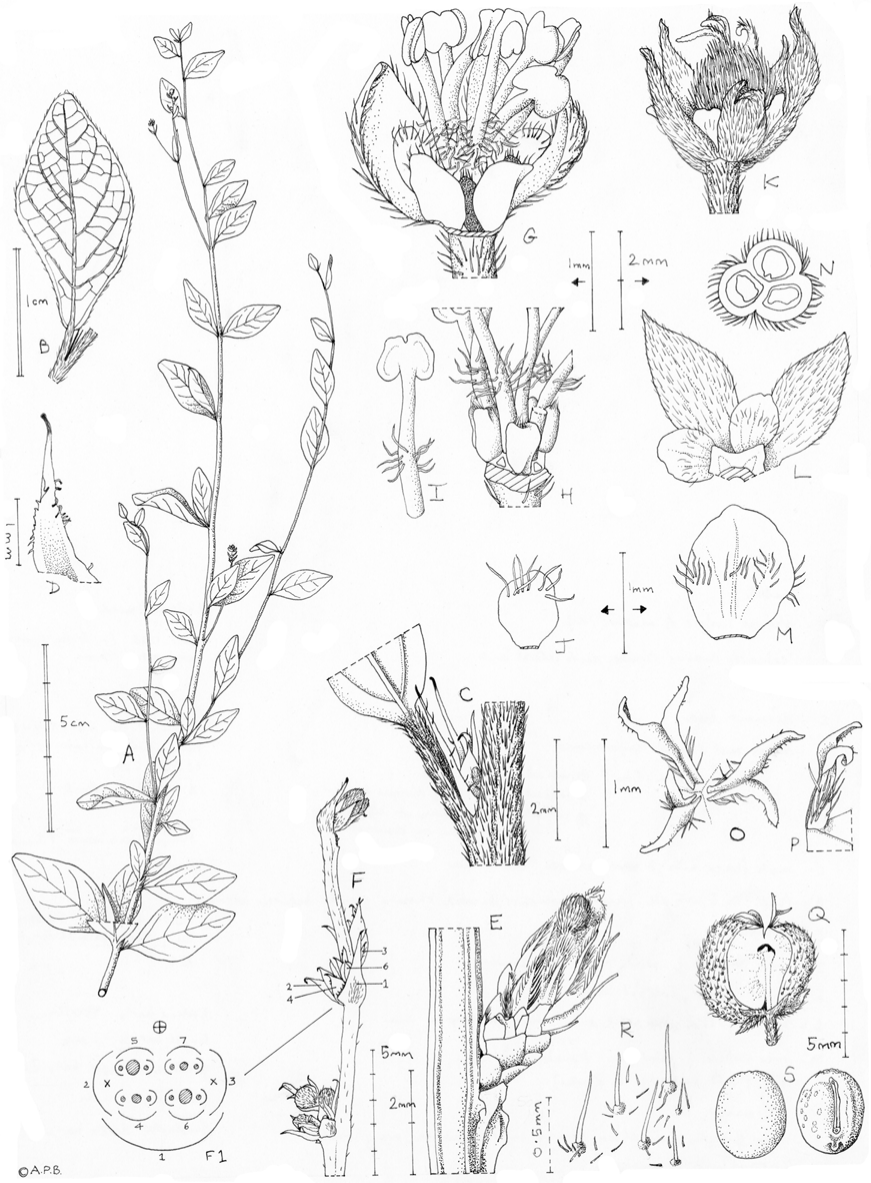
*Karima scarciesii*. A. habit, leafy stems with male inflorescences, B. leaf from near apex of primary axis, abaxial surface, C. node from near apex of primary axis showing base of leaf, stipules and incipient spur shoot, D. stipule, with glandular hairs at margin, E. leafless (dormant) spur shoot clothed mainly in persistent stipules, F. male inflorescence, F1. diagram of dissected inflorescence node/glomule: bract (1), bracteoles (2, 3), lower order bracteoles (4,5,6,7), all cross-referenced to F where visible), flower buds (cross-hatched circles), pedicels of post anthetic flowers (solid black circles); G. male flower newly anthetic (filaments not yet fully extended), proximal sepal lobes removed to expose petals, disc glands and base of stamens, H. male flower, as G, with sepal lobes and petals removed to show disc glands and filaments, I. stamen, J. petal, male flower, adaxial view, K. female flower at anthesis, proximal sepal lobe damaged, L. female flower, portion, in plan view, pistil removed (cross-hatched) showing part of the pentagonal disc, two petals and two sepal lobes, M. petal, female flower, adaxial view, N. transverse section of ovary showing uniovulate locules, O. style and stigmas, plan view, P. style-stigma, side view, Q. fruit, side view with one coccus fallen, exposing axile placented column, R. fruit wall indumentum:tubercule based bristle hairs, S. seed, dorsal (left) and ventral (right) views. Drawn by Andrew Brown from *VBS* 1223 (A), *Momoh* 94 (B-D, F-J, O-S); *Enti & Agyakwa* s.n. (E), *Jordan* 861 (K-N).

Monoecious shrub, indumentum simple, hairs sometimes gland-tipped. Stems with sparingly branched erect principal axes, and extremely short, lateral spur stems. Leaves alternate, simple, entire, nervation pinnate with the basal nerve pair acute, glandular tissue absent; petiolate; stipules persistent, becoming indurated, margins glandular-hairy when young. Inflorescences terminal on spur (short lateral) shoots; male inflorescences several-flowered, glomerulate, less usually spike-like, bracts enclosing bracteoles and multiple contracted cymules, flowers emerging sequentially; female inflorescences 1-flowered. Male flowers densely hairy, pedicellate, calyx imbricate, campanulate, divided by 2/3 into 5 ovate-elliptic concave lobes, porrect. Petals 5, alternating with sepals, included in calyx, elliptic, glabrous, except for a transverse line of hairs on adaxial surface. Disc glands 5, alternating with petals, angular-ellipsoid, erect, free, glabrous except for distal tuft of hairs. Stamens 6(–8), erect in bud, exserted, filaments terete, glabrous apart from a band of hairs above the base, anthers dehiscing laterally, connective expanded, flattened, anther cells opposite. Pollen spheroidal, inaperturate and with crotonoid exine sculpturing. Female flowers resembling but about twice the size of the male flowers, calyx divided nearly to the base into 5(–6) sepals. Petals 5(–6), twice the size of the male and visible from the exterior in the sepal sinuses; disc flattened, pentagonal; ovary shallowly 3-lobed, densely hairy; locules 3, each 1-ovulate, ovules pendulous, epitropous; styles 3, free, each bifid in distal quarter, flattened and glabrous on adaxial surface. Fruits dry, dehiscing septicidally into 3, single-seeded cocci, leaving an axile erect placenta; the seed retained in the cocci and visible through a ventral aperture, sepals and styles persistent in fruit. Seeds ellipsoid, carunculate, marbled, with a raphe line; testa membranous, translucent; tegmen mechanical, crustaceous, comprised of a single layer of subcolumellar dark brown sclereids. Endosperm white, spongy, comprising 90% of seed. Embryo with cotyledons orbicular, flattened, radicle stout, almost as long as cotyledons (Fig 3).

***Karima scarciesii*** (Scott-Elliot) Cheek comb. nov.[urn:lsid:ipni.org: names: 77153668–1]

Syn. *Croton scarciesii* Scott-Elliot ([24]:96); Hutchinson ([25]:752, [28]:296); Keay ([26]:396); Hawthorne & Jongkind ([29]:246); Berhaut ([30]:231 + figure 229, [31]:421, figure 420); Lisowski ([32]:158); Aké Assi ([33]:231); Chevalier ([34]:571); Irvine ([35]:270).

Types: Sierra Leone, Scarcies, near Mofari, fr. 12 Jan 1892, *Scott-Elliot* 4432 (syntypes, BM!, K! K000347410); Guinea, Scarcies, near Sasseni, fl. 11 Jan 1892, *Scott-Elliot* 4518 (syntype, K! K000347411). Lectotype selected here: *Scott-Elliot* 4432 (K!).

Rheophytic, fastigiate, monoecious, partially deciduous shrub 0.5–3 m, with 1-many erect branches from the rootstock. Stems 5–8 mm diam. or more at base, exudate absent or at least, not recorded. Primary axes sparingly branched, internodes 1.1–3 cm long, bearing numerous axillary spur shoots 1–6 mm long, each with 3–6 nodes, internodes extremely short. Spur shoot leaves sometimes deciduous (when submerged?), leaving dormant bud. Stems at apex terete, 0.8–1 mm diam., densely puberulent, c. 90% covered, with appressed, simple, colourless hairs (0.2–)0.4–0.7(–1) mm long, hairs straight, apices acute; indumentum persistent for several nodes, glabrescent; older stems subangular with longitudinal lines, 2.5–3 mm diam., epidermis indurated, glossy brown, lenticels not absent. Leaves densely crowded on spur shoots, more widely spread on primary axes, alternate, simple, chartaceous, stipulate, petiolate. Blades rhombic-elliptic, rhombic-ovate or obovate 1.4–1.8(–5.0) x (0.45–)0.6–0.9(–2.7) cm, apex rounded, base cuneate, abruptly rounded, margin entire, secondary nerves 4–6(–7) arising equidistantly in the distal ¾ of the midrib, equally developed, except an acute basal pair of nerves arising near the petiole apex; nerves arising from the midrib at c. 45°, arching upwards towards the margin, becoming less conspicuous and uniting with the scalariform tertiary nerves of the secondary nerve above, intersecondary and quaternary nerves inconspicuous; glandular tissue completely absent from the leaf-blade; indumentum of sparse, spreading simple hairs 0.6–1 mm long at the margin, usually hairs absent from the adaxial face and absent or very sparse on the abaxial surface, except the sparsely hairy midrib. Petiole canaliculate, 1–2(–3) mm long; indumentum as stem. Stipules persistent, subulate, rapidly becoming indurated, brown, narrowly triangular, 3–3.5 x 0.3–0.5(–1) mm, apex narrowly acute, terminating in a caducous capitate hair 0.2 mm long, margins with caduceus, sparse, capitate or glandular tipped (apices red) simple hairs 0.1 mm long, otherwise glabrous.

Inflorescences terminal on spur-shoots. Male inflorescences sessile, glomerulate, less usually spike-like, 3–19(–30) mm long, nodes 1–3, internodes 6–6.5 mm long, axis sparsely puberulent, hairs simple, as on stem; bracts naviculate (laterally compressed), ovate (2–)2.5–3.2 x 0.8–1.4 mm, margins with sparse, simple, glandular hairs; bracteoles two, ovate, 2 x 0.5 mm, resembling bracts, subtending two pairs of condensed dichasial cymes. Flowers emerging from bracts in succession, only one at anthesis at each inflorescence node, evidence of older flowers from blackened exserted pedicels. Male flowers exserted from bracts, with pedicels terete, 4 mm long, sparsely puberulent, hairs simple, spreading 0.15–0.25 mm long. Buds globose 0.9–1.2 mm diam. densely hairy. Receptacle not conspicuous at anthesis. Calyx green-white campanulate, deeply 5-lobed, 2.5–3 x 2.2–2.4 mm, lobes ovate-elliptic, erect, opening slightly at anthesis, concave, 1.3–1.6 x 0.8 mm, imbricate in bud, the two outermost lobes overlapping the bud apex, apices acute to obtuse, outer surface densely pubescent (90–100% covered) with matted, translucent, subappressed, straight, simple hairs, 0.2–0.6 mm long, inner surface sparsely hairy. Petals 5, concealed within calyx at anthesis, green-white, elliptic to obovate, 0.7–0.8 x 0.6–0.7 mm, apex rounded, base 3 mm wide, obtuse, inner surface with transverse line of hairs 0.5–0.6 mm from the base (¾ length of petal), hairs simple, 0.25–0.4 mm long. Disc purple-black, units 5, angular-ellipsoid, erect, 0.5 x 0.3 x 0.25 mm, apex rounded, truncate or slightly retuse, with a tuft of minute hairs 0.1 mm long; Stamens free, 6–7, exserted in cluster at anthesis, erect in bud; filaments white, erect, 1.2–3.5 mm long, terete, glabrous except for a band of 6–15 hairs inserted 0.5–0.7 mm from the base, hairs patent, crinkled, 0.2–0.3 mm long; anthers pale green, dorsiventrally flattened, 0.4–0.5 x 0.5 mm, dehiscing laterally, the two cells opposed, connective dilated, flattened; pistillode absent or inconspicuous.

Female inflorescences terminal, usually on separate spur branches from the male ones, peduncle 1–1.5 mm long, 1-flowered. Female flowers inconspicuous among the leaves, globose, <3.5 mm diam. Pedicel 1–1.5 mm long, moderately densely pubescent, hairs 0.1–0.6 mm long, simple, appressed to spreading. Calyx green, divided nearly to the base into 5(–6) lobes, lobes slightly concave, ovate-elliptic, to lanceolate, 3–3.4 x 1.25–1.5 mm, apex acute, base obtuse, outer surface densely hairy, hairs as male sepals, inner surface moderately hairy, as male sepals. Petals 5(–6), colour not noted, clearly visible within the sepal sinuses at anthesis, broadly elliptic to orbicular c. 1.25 x 1.1 mm, apex rounded, base obtuse, glabrous except for a transverse line of hairs on the inner face equidistant from base and apex, hairs 0.2–0.6 mm long. Disc flat, pentagonal, 1.8 mm diam., lobes 5, triangular, 0.3–0.4 x 0.7 mm, radiating from a torus 0.2–0.3 mm wide, glabrous. Stamens absent. Pistil globose, 1.8–2 mm diam., the 3 lobes (Fig 3N) densely covered in simple hairs 0.5–0.7 mm long. Locules 3, each uniovulate. Styles 3, free at base, each 1–1.3 mm long, 0.1 mm wide, bifurcate, 0.7–0.8 mm from the base, the adaxial face flattened, glabrous, with a central longitudinal groove, the abaxial surface convex, hairy, hairs simple 0.1–0.3 mm long, spreading; the style arms diverging from each other at 90°, 0.5 mm long, apex acute, adaxially glabrous, abaxially minutely puberulent, hairs 0.03 mm long, simple. Fruits green, globose, 4–5 mm diam., shallowly 3–lobed, outer surface moderately densely covered in flattened tubercles, each with a single apical, simple bristle hair, and numerous more basal simple minute hairs; sepals and styles persistent, but not accrescent. Dehiscing septicidally into 3 equal cocci, leaving a central axile vascular placenta (Fig 3Q). Cocci with ventral aperture, retaining and exposing the single seed. Seeds ellipsoid, 3.5–4 x 2–3 x 3 mm, dull, marbled, pale purple and brown, glabrous. Raphe c. 2.5 mm long, extending longitudinally from the hilum; caruncle yellow, orbicular, 0.5 mm diam., appressed to testa, inserted near the hilum. Seedcoat with membranous, translucent testa (outer seed integument), the tegmen (inner integument) mechanical, of a single layer of columnar sclereids. Endosperm white, spongy, filling 80–90% of the seed coat. Embryo flattened, 0.1–0.2 mm thick, cotyledons suorbicular, 1.5 mm diam., radicle 0.75 x 0.6 mm. (Fig 3R).

### Distribution

**Fig 4:**
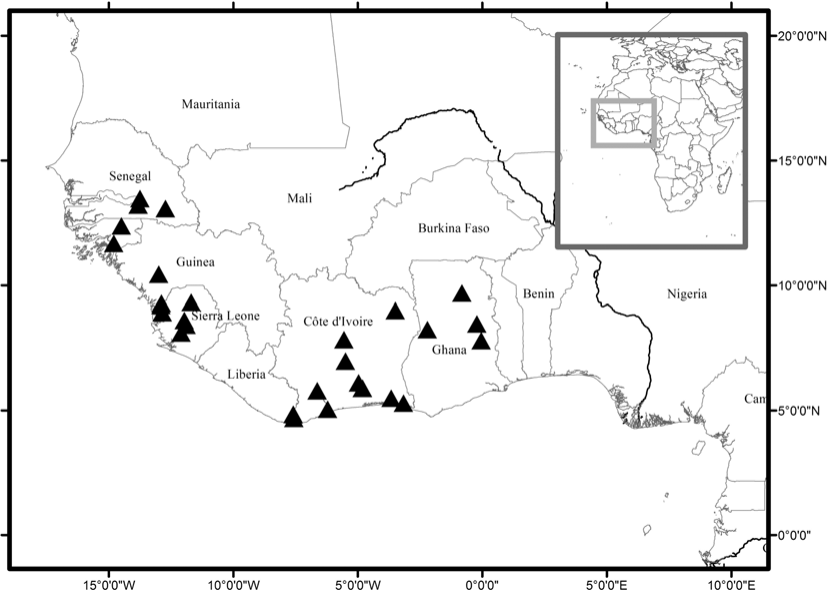
Map showing global distribution of *Karima scarciesii*. Localities are based on all known herbarium specimen records.

Scattered, along lowland rivers, Senegal, Guinea-Bissau, Guinea, Sierra Leone, Liberia, Ivory Coast, Ghana (Fig 4).

### Ecology

Totally submerged in the wet season, exposed in the dry season, with a patchy distribution, in river beds, mainly rocky, in the open, less usually on sand or silt, or in the shade (when fewer stems per plant, leaf-blades larger, petioles longer) of riverbank trees, sometimes locally common, often with other rheophytes, especially Podostemaceae and *Rotula aquatica* (Boraginaceae), usually in the evergreen forest zone (less usually in predominantly wooded grassland areas in rivers with adjoining gallery forest); *Karima* shrublets are often festooned with the epiphytic mosses *Ptychanthus striatus* and *Cinclidotus fontinatoides*; 20–250 m alt.

At study sites of patches of *Karima* at the Taia river (S2 Appendix), plants occur at densities of 24–78 per 100 m², with spacings between clumps of plants between 20–400 cm. See detailed quantitative and qualitative ecological field data in S2 Appendix.

### Local names and uses

Taduɛme (Mende language, Sierra Leone) and àsá (Temne language, Sierra Leone), both according to N.W. Thompson, recorded in Burkhill [36]. No uses are recorded but we note that this species has potential applications in reducing erosion and in stabilising the banks of fast-flowing water-courses. However, Burkhill [36] also states that in northern Ghana this shrub “offers harbour for the tsetse fly”, citing *Vigne* 3864, (K).

### Etymology

From Karim meaning generous (Arabic), one of the 99 names of God, used as a name in West Africa, and especially in Sierra Leone, specifically commemorating Dr Karim, Dean of Science at Fourah Bay College, Freetown, Sierra Leone.

### Conservation

*Karima scarciesii* is known from c. 26 locations (see map, Fig 4), having an extent of occurrence (in the sense of IUCN [18]) of 890,258 km² (calculated with Geocat, Bachman et al. [37]) and an area of occupancy of c. 30 km² (calculated using the IUCN [18] advised cell size on 1 km² for riverine species, based on 30 sites being known).

The major and only known threat to *Karmia scarciesii* is from hydroelectric projects which permanently submerge locations for this species, as has happened at Kete-Krachi in Ghana (Volta Dam), more recently in Sierra Leone at the Bumbuna Phase 1 dam (Hawthorne pers. comm. 2013) and, imminently, the second phase Yiben Dam (van der Burgt pers. comm. 2014). Total inundation and therefore local extinctions are also expected shortly to happen in Cote D’Ivoire, at Sassandra River, Chutes de Soufré, and at Chutes de Naoua, also at the Black Volta River at Bui, Ghana. It is likely that in future further hydroelectric projects will destroy additional locations for this species. Despite these threats, the taxon is here assessed as Near Threatened since the number of locations thought to survive exceeds the threshold of 10 usually required before an assessment of threatened is made with Criterion B of IUCN [18]. A detailed study has not been made of the survival of the species at its historical sites. Should this be done it is quite possible that *Karima scarciesii* might be shown to have been lost at >30% of these sites in the last 90 years, in which case, should the generation time be found to be as long as 30 years, the taxon would be eligible for reassessment as Vulnerable under Criterion A of IUCN [18]. There is insufficiently detailed population data to make IUCN assessments under either Criterion C or D.

### Additional specimens examined

SENEGAL. Banks of the Gambia River, (fide Berhaut [30,31]); Goloumbo, Sep 1951, Berhaut 765 (P 05603292!); ibid, 12 Sep 1953, Berhaut 3008 (P 05603291!); Berhaut 4105; Thiès-Diourbel, Kaolak, Niokolo-Koba, 7 Jan 1954, Berhaut 4512 (P 05603289!); Ouassadou, 1 Jan 1954, Berhaut 4298 (P 05603290!); Niokolo-Koba, 12 Jun 1958, J.G. Adam 14343 (IFAN, P 05603293!); de Tambacounda à Kédougou, Pont sur le Niri Ko, fr. 22 Jul 1989, Vanden Berghen 8749 (BR!); ibid 8755 (BR!).

GUINEA BISSAU. Chitole Region, Cusselinta, Rio Corubal, fl. fr. 10 Dec 1952, Espirito Santo 3172 (K!, MO 2572988!, P 05603295!); Gabu, Sonaco, fl. 3 Nov 1955, Espirito Santo 3585 (BR!, LISC, MO 2429504!, P 05603294!, WAG!).

GUINEA. Kindia, Konkoure, Jun 1937, H. Jacques-Félix 1784 (P 05603296!, P 05603297!, P 05603298!).

SIERRA LEONE. Near Mange, Bure, fl. 5 Jan 1953, Jordan 851 (FBC, K!); ibid fr. 7 Jan 1953, Jordan 861 (FBC, K!); Mange, Little Scarcies River, fl. 6 Apr 1958, Hepper 2613 (FBC, K!); Rowalla, st. 23 Jul 1914, N.W. Thomas 1062 (FBC, K!); Njala, Taiya River, fl. fr. 20 Jan 1927, Dalziel 8059 (E image!, FBC, K!, US 1272874); Njala (Kori), Ndelejule, fl. 25 Jan 1957, C.T. Pyne 153 (FBC, K!, WAG!); Seli River, E of Fadugu, upstream of Yiben village, Bumbuna dam lake, fl. fr. 23 May 2014, Momoh 94 (K!, SL, WAG!); Njala, 20 Jan 1927, Deighton 505 (K!); Ndilajula, near Njala, 29 May 1927, Deighton 702 (K!); Makump, edge of river Seli, 25 Jan 1929, R.R. Glanville 135 (K!).

LIBERIA. Webbo am Cavally, Dinklage 2666 (B 100591554!); Maryland, Sandy Island in Cavally River, 4°41'N 7°34'W, 8 Apr 2000, C.C.H. Jongkind & Assi-Yapo 4992 (WAG!).

IVORY COAST. Bandama River, near Tiassalé, 4°49'W, 5°54'N, fl. 10 Dec 1958, Leeuwenberg 2138 (WAG!, K!, P 05603224!); Nzi River (tributary of the Bandama), at the Ndoue-Singrobobridge, fl. 6 Nov 1961, J.J.F.E. de Wilde 3238 (K!, P 05603225!, WAG!); Bandama R., Lamto, 6 Mar 1965, Aké Assi 7969 (UCJ); ibid 26 Nov 1987, Gautier 687 (Z); Réserve de Bouna, 7 Jul 1958, Aké Assi 4995 (UCJ); Comoé River, Alépé Malamalalasso, 6 Mar 1907, Chevalier 17513 (P 05603223!, P 05603227!); Bas-Comoé, Abiati, 7 Mar 1907, Chevalier 17539 (P 05603218!, P05603219!, P0560320!); Sassandra River, Chutes de Soufré, 29 Dec 1955, Aké Assi 4012 (UCJ); ibid. Chutes de Naoua, 26 Jan 1975, Aké Assi 127089 (UCJ). Louga, near Sassandra river, fl. 25 Jan 1975, de Koning 5213 (BR!, E, MO!, WAG!); Bank of the Cavally River near Tiboto, 4°51´N 7°35´W, fl. fr. 12 Dec 1997, Jongkind 4208 (MO, UCJ, WAG!); Tiassalé-Bandama, 25 Aug 1956, J.J.F.E. de Wilde 445 (WAG 179760!); near the confluence of the Comoé river and the Kongo river, fl. 22 Apr 1968,Geerling & Bodkam 2633 (BR!, MO 2476521!, UCJ, WAG!); Sassandra, Langa, near Sassandra river, 5°3'N 6°13'W, 25 Jan 1975, de Koning 5213 (WAG!); [no locality], 3 Jan 1953, Portères 240 (P 05603217!); [no locality], 3 Jan 1953, Portères 725 (P 05603216!, P 05603221!); Ahouaty, fl. 7 Mar 1982, César 1546 (P 05603222!).

GHANA. Black Volta River at Bui, fl. 18 Aug 1964, Enti & Agyakwa in GC 35141 (GC, K!); Kete-Krachi, date unknown, VBS 1223 (GC, K!); Pong Tamale, Jun 1935, C.Vigne 3864 (GC, FHO, K!); Bui N.P., Black Volta River, Hall & Swaine 46232 (MO 2577262!); River Daka at Ekumdipe, Salaga to Kpandai, 12 July 1970, J.G. Hall s.n. (MO 2047380!).

### Morphological variation

Material illustrated from Senegal in the extreme western part of the range by Berhaut [30, 31] is atypical in having much larger leaf-blades than is normal elsewhere within the range, and also unusual crotonoid inflorescences. These are bisexual with the female at the base of the raceme, the remaining flowers being male (instead of having separate male and female inflorescences). This material merits further study since it may be a second species of the genus rather an interesting local variant.

## Supporting Information

S1 Appendix Species sampled with GenBank accession numbers for *rbcL* and *trnL-F* sequences, respectively. Voucher information is given for newly sequenced species (bold-faced).
(DOCX)

S2 Appendix Field Observations of *Karima scarciesii*. (DOCX)
