# Supplementary material for: Discovering Karima (Euphorbiaceae), a New Crotonoid Genus from West Tropical Africa Long Hidden within Croton
Source: PLoS One. 2016 Apr 6;11(4):e0152110. doi: 10.1371/journal.pone.0152110 (PMC4822767; doi:10.1371/journal.pone.0152110)
Supplement: S1 Appendix — Voucher information is given for newly sequenced species (bold-faced). (DOCX) [file pone.0152110.s001.docx]

**S1 Appendix . Species sampled with GenBank accession numbers for *rbcL* and *trnL-F* sequences, respectively.**

Voucher information is given for newly sequenced species (bold-faced).

*Aleurites moluccana* (L.) Willd., AY794883, AY794709. *Baliospermum montanum* (Willd.) Müll. Arg, AY794884, AY794726. *Baloghia inophylla* (G. Forst.) P.S. Green, AY794880, AY794707. *Bertya rosmarinifolia* Planch., AY794878, AY794705. *Beyeria leschenaultii* (DC.) Baill., AY794879, AY794706. *Blachia siamensis* Gagnep., AY794888, AY794727. *Cavacoa aurea* (Cavaco) J. Leonard, AY794889, AY794718. *Codiaeum variegatum* (L.) Blume, AY788169, AY794729. *Croton alabamensis* E.A. Sm. ex Chapm. var. *alabamensis,* AY788171, AY794692. *Crotonogyne sp.,* AY794893, AY794717. *Dodecastigma amazonicum* Ducke, AY794885, AY794711. *Domohinea perrieri* Leandri, AY794895, AY794720. *Fontainea venosa* Jessup & Guymer, AY794881, AY794708. *Garcia nutans* Vahl ex Rohr, AY794890, AY794714. *Givotia madagascariensis* Baill., AY794891, AY794715. *Hylandia dockrillii* Airy Shaw, AY794882, AY794710. *Jatropha integerrima* Jacq., AY794902, AY794685. ***Karima scarciesii*** (Scott-Elliot) Cheek, Ivory Coast, Cavally River, Jongkind 4208 (WAG), **KU213662, KU213659**. Sierra Leone, Seli River, Momoh 94 (K), **KU213661, KU213658**. *Manniophyton africanum* Mill. Arg., AY794886, AY794712. ***Neoholstia tenuifolia*** (Pax) Rauschert, Tanzania, Mtwara, Masasi, Bidgood 1889 (K)*,* **KU213663, KU213660**. *Nealchornea yapurensis* Huber, AY794865, AY794662. *Neoboutonia mannii* Benth. & Hook. f., AY794896, AY794723. *Ostodes paniculata* Blume, AY794900, AY794725. *Paracroton zeylanicus* (Mill. Arg.) N. P Balakr. & Chakrab., AY794894, AY794719. *Pausandra martini* Baill., AY794887, AY794713. *Ricinocarpos tuberculatus* Mill. Arg., AJ418817, AY794704. *Ricinodendron heudelotii* (Baill.) Heckel, AY794892, AY794716. *Sagotia racemosa* Baill., AY794903, AY794687. *Strophioblachia fimbricalyx* Boerl., AY794901, AY794728. *Tannodia cordifolia* (Baill.) Baill., AY794897, AY794721. *Trigonostemon verrucosus* J.J. Sm., AY788192, AY794703. *Vernicia montana* Lour., AY794899, AY794724.
