# Supplementary material for: Discovering Karima (Euphorbiaceae), a New Crotonoid Genus from West Tropical Africa Long Hidden within Croton
Source: PLoS One. 2016 Apr 6;11(4):e0152110. doi: 10.1371/journal.pone.0152110 (PMC4822767; doi:10.1371/journal.pone.0152110)
Supplement: S2 Appendix — (DOCX) [file pone.0152110.s002.docx]

**S2 Appendix. Field Observations of *Karima scarciesii*.**

**Njala Campus Riverbank**

Field observations of *Karima scariesii* at the above site began on May 25, 2015 on the right bank of River Taia as it flows by the Njala University campus en route to the Atlantic Ocean (see Table 1 for GPS coordinates). On a stretch of shaded riverbank, clumps of the species were located in a plot measuring 2.5 m x 40 m. The water level in the river had risen at this time of year since the wet season had begun. Some of the plants were already visibly covered by the flowing river. The bank descends into the river at an angle of 38^0^, and a 2.5 m stretch of the bank had collapsed exposing underlying clay and metamorphic rocks of the lateritic type. The distance between the ledge of the bank and the first *Karima* plant was 3 m, but there were visible signs to indicate this portion of the riverbank gets submerged at the height of the rainy season. This portion of the riverbank was also heavily shaded, and typical riverine tree species recorded included *Cynometra afzelii*, *Pterocarpus santalinoidess*, *Uapaca* sp. and two introduced species (*Hevea brasiliensis* and *Artocarpus* sp.). The ground flora was dominated by *Culcasia* sp., and *Osmunda regalis* growing on the metamorphic rock. The overlying soil was a mix of clay loam with lots of leaf litter on the floor.

Six clumps with a total of 30 plants were recorded within the plot. Within the first clump, there were three plants measuring between 15–71 cm in height, and one of the plants had started branching and producing new stems at ground level. The height of each stem was approximately 1.14 m, but the point at which each of the stems arose from its mother stem was between 11–16 cm above the substrate level. One of the plants in the plot had a circumference of 10 cm at ground level and had numerous stems which drooped over the water. The total length of this plant measured horizontally was 1.1 m. Out of the 30 plants observed in the plot, only 6 were in flower and 2 of these also had fruits on them.

**Riverbank and “Rocky Islands” (near Mogbamu village)**

Located some 20 km downstream of Njala campus, four plots (10 m x 10 m) were measured out and sampled where other patches of *Karima* occurred, one plot near a riverbank and 3 other plots on “rocky islands” in the middle of the Taia River (see Table 3 for GPS coordinates).  On the 3 rocky islands, the plants grew as though wedged between rocks (where two or three rocks are juxtaposed), or directly on the rocks wherever crevices or cracks had developed. The plants were also observed growing in depressions between rocks where sand accumulates. The plants get submerged at the height of the rainy season, bending in the direction of the flow of the river current, but are exposed to desiccation when the water level falls in the dry season. In some inaccessible areas of the rapids, the plants were observed where the lower portion of the stems had already been covered with whitewater. Plants are generally distributed in sparse clumps, and distances between individual clumps can range between 20–400 cm. Heights of plants seldom exceed 1 m, ranging between 47–120 cm. On the exposed rocks, dried plants of *Ledermanniella aloides* were observed on three of the rocky islands. Scattered trees typical of riparian habitats were observed: *Cynometra* *afzelii*, *Symmeria paniculata*, *Pterocarpus santalinoides* and *Hymenocardia lyrata* all growing wedged between rocks or partly covered at the root bases by sand deposits.

At the Mogbamu site, the riverbank was heavily shaded by trees similar to those observed on the rocky islands and the riverbank at Njala campus, with a profusion of entangled lianas in the tree canopy. The ground flora was virtually absent, except for scattered individuals of *Karima* and a lot of leaf litter on the floor. The soil was sandy to a depth of over 33 cm with streaks of mud and silt. A small creek, that flows mostly in the rainy season empties into the river, and at its mouth are small rock boulders protruding through the sand.

There were observed morphological differences between plants growing on the rocky islands without shade, in the middle of the water and those on the tree shaded riverbanks. Leaves of plants growing on the rocks have much smaller leaves, appearing narrower (elliptic/lanceolate) than those growing in shade by the riverbanks. Such leaves measure up to 2.7 cm long and 0.7 cm wide, whereas leaves on plants under shade by the riverbank measure 5 cm long and 2 cm wide, with a petiole that is 3–4 times longer than those of plants growing on the rocky islands. Whilst plants growing on the river banks are equally subjected to submersion as those on the rocks, the effect of the water current on plants is more transient than for those on the rocks which spend more of the year under water, and are also subjected to less desiccation than those on the rocks.

Plants growing on the rocks develop profuse stem branching from the root level, where the stem branches are held above the substratum, rarely getting wider than an index finger, and with very hard bark.  A single plant on the rock can develop between 4–56 stem branches from ground level and attain a height of up to 1.2 m. Some stems on the shrubs appear dried up with fewer leaves and are often festooned/laden with moss. Stems of plants growing at the edge of the riverbank under shade can reach a circumference of 10 cm, with bark that can be easily peeled, exposing a cream-whitish coloured inner stem.  Stems can develop at ground level but are not as profuse as on plants growing on the rocks, and their roots are not exposed as with those on the rocks.

Female flowers on stems are not always terminal, and between one and two stems were observed with between 2–5 fruits (one of which was aborted on the stem that had 5 fruits). Plants have been observed flowering, and to some extent fruiting at most sites (see Table 3).  Flowering can be profuse and most times dominated by male flowers on most stems, with fewer female flowers and fruits. Not all stems on a plant produce flowers at the same time.

Table: Plot Inventory of *Karima* scarciesii along River Taia, Sierra Leone.

| Site Description | GPS  Coordinates  Altitude | # Plants  /100 m^2^ | Height range  (cm) | # Flowering | # Fruiting |
| --- | --- | --- | --- | --- | --- |
| Riverbank in shaded area with steep slope and riparian trees: *Cynometra* *afzelii,* *Pterocarpus santalinoides*, and *Uapaca* sp. Ground flora included *Osmunda regalis* and *Culcasia* sp; clay loam soil overlying metamorphic rocks; abundant leaf litter. | N 08º06’56”  W 12º04’46”  60 m | 24 | 35–225 | 3 | 1 |
| Rocky island in the middle of River Taia; granite with occasional ferralitic intrusion and sand deposits. Typical species recorded included *Hymenocardia lyrata*, *Symmeria paniculata*, *Pterocarpus santalinoides*, *Cynometra* *afzelii.* Podostemaceae (*Ledermanniella* sp) observed on rock. | N 08º00’34”  W 12º06’29”  33 m | 53 | 48–120 | 16 | 1 |
| Sand bar generally by the riverbank, wedged between the mouth of a small creek and the river, with protruded rock boulders shaded by riparian trees like *Pterocarpus santalinoides*, *Cynometra* *afzelii* and several species of dense lianas. Soil was sandy with thin layer of mud and no indication the plants were growing on rocks. Leaves were approximately 5cm x 2cm. | N 08º00’2.0”  W 12º06’26”  49 m | 28 | 25–232 | 4 | 1 |
| A rocky island within 25m of the riverbank with a large portion of it made of igneous rocks, pool of water and sand deposits in some areas. Tree cover comprised of *Pterocarpus santalinoides*, *Cynometra* *afzelii* and *Symmeria paniculata*. Herbaceous plants included *Cynodon dactylon*, *Stachytarpheta* sp, *Ludwigia* sp and *Hygrophila odora.* | N 08º00’38”  W 12º06’30”  32 m | 78 | 48–100 | 32 | 8 |
| A denuded rocky-sand bar some 75m from the edge of the riverbank. Generally flat with most of the rocks in the process of weathering and with crevices in them, but strewn with sand. *Cynometra* *afzelii*, *Pterocarpus santalinoides*, *Hymenocardia lyrata* and *Symmeria paniculata* dominate the tree layer with several unidentified herbs/grasses. | N 08º00’26”  W 12º06’25”  27 m | 35 | 20–65 | 13 | 1 |
